# Phenolic Compounds of *Rhodiola rosea* L. as the Potential Alternative Therapy in the Treatment of Chronic Diseases

**DOI:** 10.3390/ijms241512293

**Published:** 2023-07-31

**Authors:** Jurga Bernatoniene, Valdas Jakstas, Dalia M. Kopustinskiene

**Affiliations:** 1 Institute of Pharmaceutical Technologies, Faculty of Pharmacy, Medical Academy, Lithuanian University of Health Sciences, Sukileliu pr. 13, LT-50161 Kaunas, Lithuania; jurga.bernatoniene@lsmuni.lt (J.B.); valdas.jakstas@lsmuni.lt (V.J.); 2 Department of Drug Technology and Social Pharmacy, Faculty of Pharmacy, Medical Academy, Lithuanian University of Health Sciences, Sukileliu pr. 13, LT-50161 Kaunas, Lithuania; 3 Department of Pharmacognosy, Medical Academy, Lithuanian University of Health Sciences, Sukileliu pr. 13, LT-50161 Kaunas, Lithuania

**Keywords:** *Rhodiola rosea* L., phenolic compounds, salidroside, chronic diseases, cancer

## Abstract

The roots and rhizomes of *Rhodiola rosea* L. (Crassulaceae), which is widely growing in Northern Europe, North America, and Siberia, have been used since ancient times to alleviate stress, fatigue, and mental and physical disorders. Phenolic compounds: phenylpropanoids rosavin, rosarin, and rosin, tyrosol glucoside salidroside, and tyrosol, are responsible for the biological action of *R. rosea*, exerting antioxidant, immunomodulatory, anti-aging, anti-fatigue activities. *R. rosea* extract formulations are used as alternative remedies to enhance mental and cognitive functions and protect the central nervous system and heart during stress. Recent studies indicate that *R. rosea* may be used to treat diabetes, cancer, and a variety of cardiovascular and neurological disorders such as Alzheimer’s and Parkinson’s diseases. This paper reviews the beneficial effects of the extract of *R. rosea*, its key active components, and their possible use in the treatment of chronic diseases. *R. rosea* represents an excellent natural remedy to address situations involving decreased performance, such as fatigue and a sense of weakness, particularly in the context of chronic diseases. Given the significance of mitochondria in cellular energy metabolism and their vulnerability to reactive oxygen species, future research should prioritize investigating the potential effects of *R. rosea* main bioactive phenolic compounds on mitochondria, thus targeting cellular energy supply and countering oxidative stress-related effects.

## 1. Introduction

**Figure 1:**
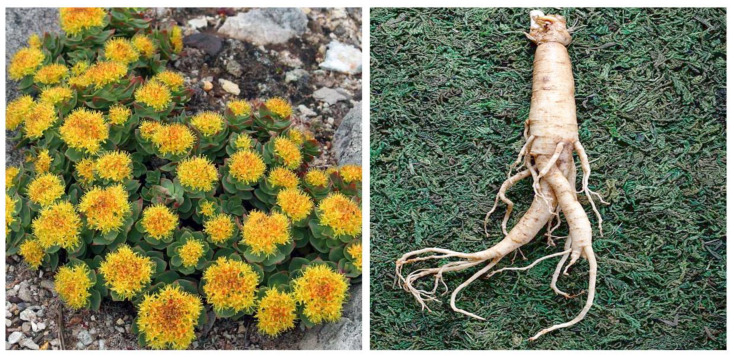
*Rhodiola rosea* L. plant and rhizomes.

*Rhodiola rosea* L. belongs to the family of Crassulaceae [1,2,3]. It is also known by the common names Golden Root, Arctic Root, and Roseroot [3]. The plant is widespread in Asia (mainly Siberia), Northern Europe, Britain, and North America where it grows on mountain rocks and cliffs at high altitudes. *R. rosea* is a perennial plant, 5–40 cm tall, it possesses a short, thick rhizome and grows in several stems from it [4,5]. It blooms in summer, the flowers are yellow to greenish yellow, with four sepals and four petals (Figure 1).

The weight of the *R. rosea* root can reach 1 kg [4,5]. *R. rosea* roots and rhizomes are continuously growing horizontal underground stems which put out lateral shoots and adventitious roots at intervals. The raw material, roots and rhizomes, are collected when seeds become mature, usually in August–September [4].

The healing properties of the plant were first time described by Dioscorides in *De Materia Medica* where it was named *Rodia riza*. Carl Linnaeus named this plant *Rhodiola rosea* as rhizomes of it had a smell similar to rose oil [3]. There are more than 200 species of *Rhodiola*, but only about 20 of them are used for medical purposes of which the most important are: *R. alterna, R. brevipetiolata, R. renulate, R. kirilowii, R. quadrifida, R. sachalinensis*, and *R. sacra*, and the most widely used, *Rhodiola rosea* [6,7].

Carl Linnaeus described *Rhodiola* to be useful for the treatment of headaches, “hysteria”, hernias, discharges, and as an astringent [3,6,7]. The use of the *Rhodiola* spp. root for healing purposes is described in the first Swedish national Pharmacopoeia in 1755. Kelly and Panossian summarized in their reviews that *R. rosea* was known as a healing plant in Scandinavia, Russia, China, and other countries [3,6]. In traditional Chinese and Northern European medicine, the root of *R. rosea* was used to increase strength [8], physical capacity [7], longevity [2,3], against fatigue [1,9], depression [3,10], to diminish anemia [6], impotence [11], infectious and viral diseases [3,12,13], and against gastrointestinal tract malfunction [11] and neurological problems [1,3,6]. It is one of the most popular tonics in Siberian medicine [3,7]. Water-based extract of *R. rosea* was used against diabetes, anemia, tuberculosis, and liver and stomach diseases in Russia and Siberia [3,7]. In Tibetan medicine, *Rhodiola* spp. were used as a tonic, also in the treatment of anemia and impotence [3,6,14]. In Middle Asia, tea of *R. rosea* is used as an effective means against cold and flu. Mongolian doctors used *R. rosea* against tuberculosis and cancer, whereas Vikings used it to increase physical strength and energy [15,16,17].

*R. rosea* traditionally was used to enhance the immune system, increase red blood cells count and hemoglobin, as an anti-inflammatory remedy, also against cancer and allergies [5,18,19]. *R. rosea* was used also for hyperglycemia treatment [5,18,19,20] furthermore, it was used as a remedy to lessen leukocytosis [4,21]. *R. rosea* is considered as an adaptogen, enhancing physical and mental states [22], stimulating ability to adapt to various environmental changes [23], increasing cellular and common resistance to harmful factors, normalizing heart blood circulation and central nervous system activities [4,24,25,26,27,28].

The bioactive constituents of *R. rosea* include alkaloids, glycosides, phenolic compounds, volatile oils, coumarins, steroids, and organic acids [29]. Among these constituents, phenolic compounds are of particular interest due to their potential therapeutic properties [30]. Seven phenolic groups: flavonoids, catechins, procyanidins, phenylpropanoids, gallotannins, ellagitannins, and anthocyanins have been quantified in *R. rosea* [30]. These phenolic compounds exhibit antioxidant properties by scavenging reactive oxygen species (ROS) and neutralizing free radicals, thereby mitigating oxidative stress and cellular damage [31]. Furthermore, they possess anti-inflammatory effects, modulating key inflammatory pathways and reducing the production of pro-inflammatory molecules [32]. Through these anti-inflammatory mechanisms, *R. rosea* bioactive compounds can potentially ameliorate chronic inflammation, a common underlying factor in various diseases. Moreover, they have been associated with reducing fatigue and restoring energy levels, which may be attributed to their adaptogenic properties, helping the body cope with stress and enhancing physical and mental performance [1,11,23,33]. These multifaceted properties of *R. rosea* phenolic compounds make them promising candidates for therapeutic interventions in conditions characterized by oxidative stress, inflammation, and fatigue-related issues. Nevertheless, further research is warranted to elucidate the exact mechanisms of action and optimize their potential applications in clinical settings.

The traditional use and clinical efficacy of different *R. rosea* preparations in managing stress-induced conditions, cognitive functions, mental performance, physical performance, and aspects of cardiovascular and reproductive health, were overviewed recently, concluding that the results provide a promising basis for the clinical effectiveness of *R. rosea* preparations in addressing various stress-related conditions and related physiological dysfunctions [11]. Furthermore, clinical and experimental studies on the anti-inflammatory benefits and mechanisms of *R. rosea* were reviewed, providing evidence and guidance for its potential use in treating various diseases like cardiovascular, neurodegenerative diseases, diabetes, sepsis, and cancer [32]. Recent reviews discussed the therapeutic effects of salidroside, the main bioactive compound of *R. rosea*, highlighting its potential as a drug candidate with potent antioxidant and anti-inflammatory properties [32,34,35], anticancer activity [36], beneficial for treating metabolic and cardiovascular disturbances [37,38,39], ischemic stroke [40], and CNS diseases [41].

This review focuses on the molecular mechanisms of action of *R. rosea* phenolic compounds in the treatment of chronic diseases and identification of their prospective therapeutic targets. Understanding the synergistic interactions between the various bioactive compounds in *R. rosea* can lead to the development of optimized treatment approaches, potentially offering safer and more environmentally friendly alternatives to conventional therapies and integrating traditional herbal medicine into modern healthcare practices.

## 2. Chemical Composition of *Rhodiola rosea* L.

*R. rosea c*ontains a variety of chemical compounds that contribute to its medicinal properties. The chemical composition of *R. rosea* can vary depending on factors such as the geographic origin, cultivation conditions, and extraction methods used. *Rhodiola* rhizomes contain organic acids (oxalic, citric, malic, gallic, and succinic), essential oils, fats, waxes, phenolics including tannins, sterols, glycosides, and proteins [4,5,42]. The main bioactive compounds of *R. rosea* are phenylpropanoids rosavin, rosarin, rosin, tyrosol glucoside salidroside, and tyrosol.

The essential oils found In *R. rosea* contribute to its characteristic aroma and may have additional health benefits. The composition of these essential oils can vary, but they often contain compounds such as pinene, limonene, and cinnamyl alcohol derivatives. The dried rhizomes contain 0.05% essential oil. The rose-like odor of *R. rosea* is due to geranylformate, geranyl acetate, benzyl alcohol, and phenylethyl alcohol, and the most important, geraniol. Its oxygenated metabolite rosiridol is an aglycon of rosiridin [42].

Polar compounds of *Rhodiola rosea* include monoterpene alcohols, their glycosides and cyanogenic glycosides, phenylethanoids, phenylpropanoids, flavonoids, arylglycosides, proanthocyanidins, and other gallic acid derivatives [2,5,7,43]. Biologically active compounds include phenolic and/or cyanogenic glycosides with antidepressive, anti-fatigue, cognitive-enhancing, anti-anoxia, hepatoprotective, anti-allergy, anti-inflammatory properties. The constituent with known therapeutic activity is *p*-phydroxyphenylethyl-*O*-β-d-lucopyranoside (Syn. salidroside, rhodioloside, and rhodosin) [2,5,35,44]. Proanthocyanidins constituting a fairly large portion of the *Rhodiola* extracts (ca. 30% of the 70% acetone dry crude extract), were also noted for significant bioactivities including antioxidant, anti-cancer, anti-inflammatory, anti-allergic, anti-mutation, anti-aging, and improving liver function [45,46]. *R. rosea* contains various flavonoids, such as kaempferol, quercetin, and their glycosides [46]. Flavonoids, due to their antioxidant and anti-inflammatory properties may contribute to the overall health benefits of *R. rosea* by supporting cellular health, reducing oxidative stress, and modulating inflammatory processes [46,47].

Characteristic feature of *R. rosea* is the presence of cynnamic alcohol glucosides and relatively high content of the phenylpropanoids: rosavin, rosarin, and rosin [48,49]. Salidroside was considered to be responsible for *R. rosea* adaptogenic and stimulant properties [3,10]. Therefore, liquid *R. rosea* extract was standardized according to the amount of salidroside which had to be at least 0.8%. However, salidroside was also found in other species (*Salix triandra* L., Salicaceae) such as in *Vaccinium vitis-idaea* L., Ericaceae, and *Rhododendron* L., Ericaceae. The highest concentration of salidroside was found in many *Rhodiola* plant species. However, chemical composition of *R. rosea* differs from other *Rhodiola* species [45]. Phenylpropanoids rosavin, rosarin, and rosin are specific compounds found only in *R. rosea* [3,45]

**Figure 2:**
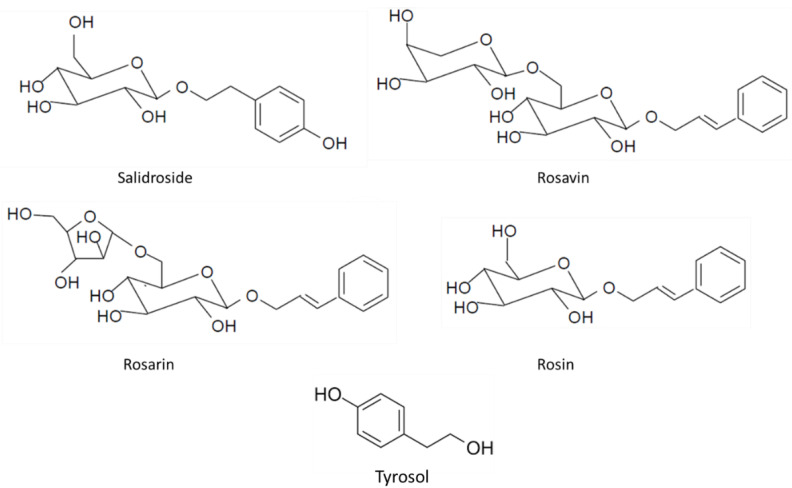
Chemical structures of main bioactive compounds from rhizomes of *Rhodiola rosea* L.

Salidroside is a glucoside of tyrosol (2-(4-hydroxyphenyl)ethyl-β-d-glucopyranoside). Rosavin ((2*E*)-3-Phenylprop-2-en-1-yl α-l-arabinopyranosyl-(1→6)-α-d-glucopyranoside), rosarin ((2*E*)-3-Phenylprop-2 en-1-yl α-l-arabinofuranosyl-(1→6)-β-d-glucopyranoside), and rosin (trans-cinnamyl *O*-β-d-glucopyranoside) are cinnamyl alcohol glycosides [4,5,18,35,45]. Their chemical structures are shown in Figure 2.

In contrast to many natural compounds, salidroside is water-soluble and highly bioavailable via oral administration [18]. In experiments with rats, salidroside was rapidly absorbed when administered orally, and eliminated through kidney via urine excretion [50,51]. The oral bioavailability depended on the dosage within the range of 32–98% [50,51]. When administered intravenously, 64% of salidroside was excreted via urine, while only 23.80% of salidroside was excreted after oral administration [50,51]. Salidroside itself was distributed mainly into fat, ovary, testis, and skeletal muscle. Salidroside undergone extensive metabolism in liver, where it was converted to its aglycone p-tyrosol in liver, and then distributed to various tissues [50,51].

Since phenylpropanoids rosavin, rosarin, rosin, salidroside, and tyrosol are the main active compounds of *R. rosea*, responsible for it biological activity [3,4,5,18], *R. rosea* crude drugs are standardized according to the amount of rosavin (not less than 3%) and salidroside (0.8–1%) [2,3,4,5,18], the proportion of these compounds in *R. rosea* rhizomes is also 3:1 [2,3,4,5,18].

## 3. Toxicity of *Rhodiola rosea* L. Preparations

It should be noted, that it is challenging to achieve consistent effectiveness over time in herbal medicinal products; therefore, the safety and effectiveness observed in one brand or extract may not necessarily apply to others [52]. *R. rosea* was classified as an adaptogen with no severe adverse effects reported, but limitations have been highlighted due to fragmented research designs [8]. *R. rosea* is safe according to animal toxicology tests. Rhodiola species, including *R. rosea*, showed neither acute nor chronic toxicity within therapeutic windows. Furthermore, salidroside, a primary active component of *R. rosea*, was not genotoxic in mice or maternal or embryonic toxic in rats at doses of 0.5, 0.25, and 0.125 g/kg [4]. However, despite being generally considered safe, *R. rosea* extract exhibited cytotoxic effects in cultured primary cortical neurons at a concentration of 100 μg/mL [53]. *R. rosea* extract WS^®^ 1375 was found to be safe and generally well-tolerated in subjects with life-stress symptoms, adverse events were mostly of mild intensity, and no serious adverse events were reported [54].

Compared with ginseng and other adaptogens, *R. rosea* is less toxic. LD_50_ of dry *R. rosea* extract for rats is 3.36 g/kg, the equivalent dosage for humans weighting ~70 kg is 235 g [4,5], and as a usual dosage is ~600 mg daily, there is a big safety margin. For sportsmen and during intense brainwork, 3 times higher doses can be used [3,4,5,55].

*R. rosea* containing supplements should be used in the first part of the day. After prolonged use of higher doses of *R. rosea* (1.5–2 g), symptoms of irritability and insomnia may be noted [3,6]. If irritability and/or headaches, and/or insomnia and/or agitation, and/or nervousness develop, *R. rosea* use should be suspended or the dosage decreased [3,6]. *R. rosea* supplements should not be used in the case of irritability, fever, or during the latter part of the day, as insomnia can develop [3,6].

## 4. Pharmacological Activity and Mechanism of Action of *Rhodiola rosea* L. Extract and Its Main Constituents in Chronic Diseases

Chronic diseases, also known as non-communicable diseases, are long-term health conditions that typically progress slowly and persist over an extended period [56]. Unlike acute illnesses that have a sudden onset and short duration, chronic diseases tend to be ongoing and may require long-term management and care. The most common chronic diseases include cardiovascular diseases, e.g., heart disease and stroke; cancer; chronic respiratory diseases, e.g., chronic obstructive pulmonary disease and asthma; diabetes; obesity; neurological disorders, e.g., Alzheimer’s disease and Parkinson’s disease; depression; anxiety autoimmune disorders, e.g., rheumatoid arthritis and multiple sclerosis; chronic kidney disease; liver disease, and many others [56]. Chronic diseases, influenced by a combination of genetic, behavioral, environmental, and socio-economic factors, are a significant global health concern [57]. Managing chronic diseases often involves long-term treatment, lifestyle modifications, and regular monitoring in order to control symptoms, slow disease progression, prevent complications, and improve the quality of life for patients living with these conditions [57]. *R. rosea* potential to alleviate stress, reduce fatigue, and improve cognitive function can enhance overall well-being and quality of life of patients with chronic diseases [31,33]; therefore, *R. rosea* supplements may have potential benefits as an adjunctive therapeutic approach.

**Figure 3:**
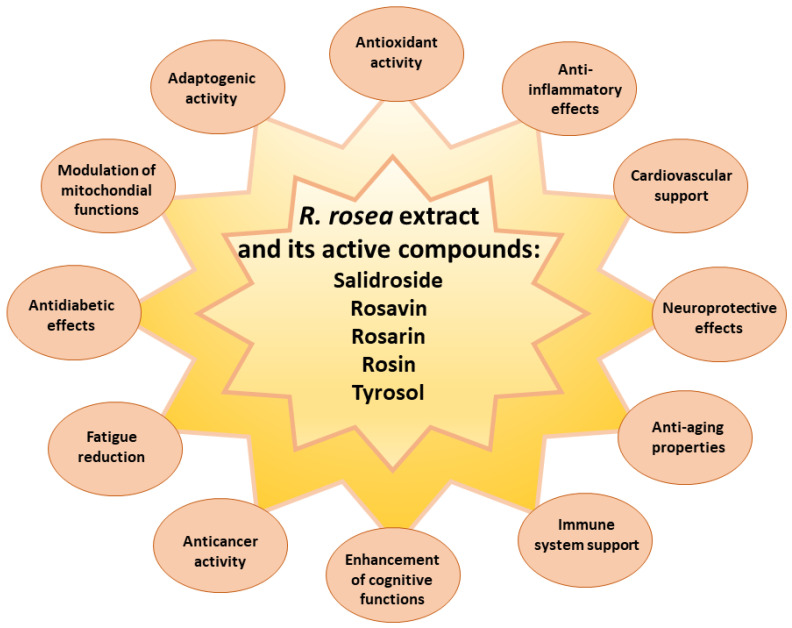
Main biological activities of *Rhodiola rosea* L. and its bioactive compounds.

Pharmacological studies of *R. rosea* extract and its main bioactive compounds have demonstrated adaptogenic, anti-fatigue, and anti-stress (neuroprotective, cardioprotective, and hepatoprotective) effects, antioxidant effects, stimulation of the central nervous system enhancing cognitive functions: attention, learning, and memory [1,2,3,4,5,18], antidepressant and anxiolytic effects, normalizing endocrine activity and increasing life-span [6,58], also anti-cancer effects and diminished symptoms of Parkinson disease [4,15,19,59,60]. *R. rosea* decreases the level of catecholamines produced by adrenal glands during stress and indirectly impairs their release, and decreases cAMP level in the myocardium [24,25,26,27,28,61]. Antiarrhythmic effect can be due to the synthesis of opioid peptides and stimulation of central and peripheral opioid receptors [61]. The main beneficial effects of *R. rosea* and its bioactive compounds are summarized in Figure 3.

### 4.1. Rhodiola rosea L. and Its Main Constituents in Neurological Disorders

*R. rosea* has potential effects in neurological disorders, including neuroprotection, cognitive enhancement, mood regulation, anti-inflammatory actions, and modulation of neurotransmitters [40,41,58,62]. Adaptogenic, cardioprotective, and stimulating central nervous system effects are attributed to the activation of biogenic monoamines (serotonin, dopamine, and norepinephrine) in the cortex, brain stem and hypothalamus, and stimulation of opioid peptides (β-endorphins) [4,58]. In the experiments with rats treated for 10 days with *R. rosea* extract, the level of norepinephrine and dopamine decreased in the cortex and brain stem, increased in the hypothalamus, and the level of serotonin changed in the opposite direction. It is suggested that *R. rosea* inhibits activities of monoaminoxidase and catechol-O-metyltransferase, responsible for the degradation of monoamines thus having effect on their levels. Also, it is supposed that *R. rosea* lessens the transport of neuromediators in the brain [1,4,9]. *R. rosea* active compound rosiridin inhibited monoamineoxidases A and B in vitro, thus acting against depression and senile dementia [63].

The neuroprotective activity of salidroside mainly is attributed to the decreased oxidative stress and increased antioxidant enzymes, Nrf2/HO-1 pathways, decreased inflammation through suppression of NF-κB pathway and PI3K/AKT pathways [64].

*R. rosea* antioxidant and anti-inflammatory activities may help to reduce oxidative stress and inflammation, which are implicated in the development and progression of conditions like Alzheimer’s disease, Parkinson’s disease, and stroke [58]. Parkinson’s disease is characterized by the decreased amount of substantia nigra dopaminergic neurons which causes reduced striatal dopamine levels [62]. *R. rosea* and its active compounds can help to alleviate the disease at the cellular level by decreasing microglia activation, attenuating damage from radical oxygen species, supporting correct protein folding, chelating iron, increasing the substantia nigra blood flow, and promoting dopaminergic cell growth [62]. In the Parkinson’s disease model in vitro, 1-methyl-4-phenyl-pyridinium (MPP^+^)-injured SN4741 cells, salidroside pretreatment improved cellular viability, inhibited apoptosis, and restored the mitochondrial membrane potential and complex I activity via regulation of the mitochondrial myocyte enhancer factor 2D (MEF2D), NADH dehydrogenase 6 (ND6) pathway [65]. Also, salidroside protected mitochondrial complex I activity, dopaminergic neurons, and preserved normal behavior in the Parkinson’s disease model in vivo in 1-methyl-4-phenyl-1,2,3,6-tetrahydro-pyridine (MPTP)-lesioned mice [65]. Salidroside was demonstrated to exert neuroprotective effects in vitro by enhancing PINK1/Parkin-mediated mitophagy in MPP^+^/MPTP-induced Parkinson’s disease models and preserving mitochondrial Complex I activity [66]. Furthermore, salidroside decreased the MPP^+^/MPTP-induced decline in cell viability, blocking the increase in reactive oxygen species (ROS), malondialdehyde, and 8-hydroxy-deoxyguanosine levels and upregulating the superoxide dismutase, catalase, glutathione peroxidase, and glutathione levels [67]. Furthermore, salidroside preserved Complex I activity via DJ-1/Nrf2-mediated antioxidant pathway [67]. Salidroside protected neurons in a Parkinson’s disease model in SH-SY5Y cells via the preservation of autophagy, which attenuated the phosphorylation of pathological α-synuclein in neurons predominantly via mTOR/p70S6K but not the PI3K/Akt signaling pathway [68]. Moreover, salidroside can induce rat mesenchymal stem cells to differentiate into dopaminergic neurons [69]. Also, salidroside enhanced α-synuclein clearance via ubiquitin-proteasome system in SH-SY5Y cells [65]. In MPTP/MPP^(+)^ models of Parkinson’s disease, salidroside pretreatment protected dopaminergic neurons by reducing the production of ROS-NO, regulating the ratio of Bcl-2/Bax, inhibiting cytochrome-c and Smac release, and suppressing caspase-3, caspase-6, and caspase-9 activation, and α-synuclein aggregation [70].

Alzheimer’s disease is characterized by deposits of aggregated amyloid-β (Aβ) peptide and neurofibrillary tangles in the brain parenchyma as well as changes in behavior and cognitive impairment [58]. Salidroside extracted from *R. rosea* showed protective effects against Aβ-induced neurotoxicity in a Drosophila Alzheimer’s disease model [71] as well as in APP/PS1 mice Alzheimer’s disease model, where behavioral performance was improved, the soluble and insoluble Aβ levels decreased, levels of synapse-related proteins increased and, and PI3K/Akt/mTOR signaling upregulated [72]. Primary cultured neurons treated with Aβ oligomers *R. rosea* extract and one of its main constituents, tyrosol, significantly inhibited Aβ oligomer-induced caspase-3 activation [73]. In Alzheimer’s disease model 5XFAD of transgenic and non-transgenic mice, tyrosol alleviated synaptic disturbance, and improved oxidative stress responses and cognitive impairment [73]. Salidroside protected PC-12 cells against Aβ-induced apoptosis by activation of the ERK1/2 and AKT signaling pathways [74].

Thus, *R. rosea* exhibits neuroprotective effects by reducing oxidative stress and inflammation, potentially slowing down the progression of neurological disorders [58]. It also enhances cognitive function, including memory, attention, and learning abilities, which can benefit conditions associated with cognitive decline [63]. Additionally, *R. rosea* regulates mood by modulating neurotransmitters and neuroendocrine pathways, offering potential relief for mood disorders [75,76]. Its anti-inflammatory properties may help reduce neuroinflammation in conditions like multiple sclerosis and neurodegenerative disorders [77,78,79,80]. Furthermore, *R. rosea* modulates neurotransmitter levels, such as serotonin, dopamine, and norepinephrine, which play a crucial role in regulating mood, cognition, and motor function in neurological disorders [4,58]. The mechanisms of these effects may include the inhibition of monoamine oxidase, leading to increased availability of serotonin, dopamine, and norepinephrine, potentially enhancing mood, the influence on serotonin receptors, impacting mood regulation, and affecting dopamine levels, contributing to improved mental well-being, the stimulation of the release of beta-endorphins, providing stress relief, and indirect protection of neurons resulting in the changes in neurotransmitter levels due to *R. rosea* antioxidant properties [4,58]. However, more extensive and well-controlled studies are needed to fully understand its mechanisms of action and potential benefits.

### 4.2. Rhodiola rosea L. and Its Main Constituents in Cardiovascular Diseases

Cardiovascular diseases encompass a range of medical conditions affecting the heart and blood vessels, including coronary artery disease, hypertension, heart failure, arrhythmias, valvular heart disease, cardiomyopathy, and stroke [81]. These disorders can range from mild to severe and can have significant implications for overall cardiovascular health [81].

*R. rosea* has shown several beneficial effects on the cardiovascular system [5,82]. Studies have indicated its cardioprotective properties, demonstrating its ability to reduce oxidative stress and inflammation in the heart [4,82]. The effects of salidroside (20 mg/kg or 40 mg/kg for 14 days) from *R. rosea* L. on myocardial ischemia in male Sprague–Dawley rats were investigated, resulting in reduced ST-segment elevation and decreased levels of biomarkers associated with myocardial damage, inflammation, and oxidative stress [83]. Salidroside also increased antioxidant activity and suppressed the expression of proteins related to oxidative stress and inflammation [83]. Furthermore, the cardioprotective effects of salidroside (were investigated in a mouse model of myocardial infarction, where salidroside was provided at 200 mg/kg/day i.g. for 21 days [84]. It reduced mortality, improved cardiac function, attenuated myocardial remodeling, and promoted angiogenesis, decreasing inflammation and apoptosis [84].

Furthermore, *R. rosea* has been studied for its potential to regulate blood pressure. Research has shown that it can promote blood vessel relaxation and improve blood flow, contributing to the maintenance of healthy blood pressure levels [82]. *R. rosea* main active compound salidroside has been demonstrated to decrease atherosclerotic plaque formation and inhibit pulmonary hypertension in animal models [82,85]. Additionally, salidroside has been shown to reduce blood pressure and alleviate cerebrovascular contractile activity in diabetic rats [82,85]. Salidroside has also been found to protect the cardiovascular system and improve cardiac function in rats with chronic heart failure [86]. Salidroside has also been shown to inhibit platelet function and thrombosis through the AKT/GSK3β signaling pathway, suggesting its potential as a therapeutic drug for treating thrombotic or cardiovascular diseases [85]. *R. rosea* extract has been found to attenuate pulmonary hypertension in chronic hypoxic rats and inhibit atherosclerosis formation in high-fat diet-fed rabbits [87]. Overall, *R. rosea* has shown promising cardiovascular protective effects, including its ability to inhibit platelet function and thrombosis, improve cardiac function, and protect against myocardial ischemia-reperfusion injury [34,82]. These effects are attributed to its various chemical components, such as salidroside, which have been found to have antioxidant, anti-inflammatory, and anti-aging activities [34,88]. Further research is needed to fully understand the mechanisms underlying these effects and to explore the potential therapeutic applications of *R. rosea* in cardiovascular diseases.

### 4.3. Antidiabetic Effects of Rhodiola rosea L. and Its Main Constituents

Diabetes mellitus is a metabolic condition characterized by compromised lipid homeostasis and glucose metabolism, which progressively results in persistent hyperglycemia. *R. rosea* and especially its active compound salidroside have been shown to exert antidiabetic activities in various in vitro and in vivo models of diabetes [35,60,64,89]. Salidroside can improve glucose tolerance, insulin sensitivity, and β-cell and liver functions, and inhibit adipogenesis, inflammation, and oxidative stress [37].

In a leptin receptor knockout (db/db) mouse model of type 2 diabetes, *R. rosea* extract improved fasting blood glucose levels, altered the response to exogenous insulin, and decreased circulating lipopolysaccharide and hepatic C-reactive protein transcript levels [90]. In streptozotocin-induced diabetic rats, *R. rosea* water extract improved hyperglycemia via an increase in β-endorphin secretion from adrenal gland to activate opioid μ-receptors [91]. Salidroside improved glucose homeostasis in obese mice by repressing inflammation in white adipose tissues and restoring leptin sensitivity in hypothalamus [92]. Also, salidroside ameliorated cerebrovascular vasodilation in streptozotocin-induced diabetic rats through improving the function of BKCa channel in smooth muscle cells [93]. In diabetic db/db mice salidroside significantly reduced blood glucose and ameliorated diabetic cardiomyopathy by modulating iron metabolism [94].

In diabetic db/db and high-fat diet-induced mice, salidroside suppressed reactive oxygen species production and restored mitochondrial membrane potential via reducing NOX2 expression and inhibiting JNK-caspase 3 apoptotic cascade thus protecting β-cell survival and preventing β-cell failure via AMPK activation [95]. Upregulation of AMPK signaling pathway is considered as one of the most important antidiabetic mechanisms of action of salidroside [60].

Thus, *R. rosea* exhibits several beneficial effects in diabetes management. It helps regulate blood sugar levels by enhancing glucose uptake, increasing insulin sensitivity, and promoting insulin release [37]. Moreover, *R. rosea* exerts antioxidant and anti-inflammatory actions, thereby mitigating oxidative stress and chronic inflammation commonly associated with diabetes [37]. *R. rosea* also exhibits lipid-lowering effects by decreasing total cholesterol, LDL cholesterol, and triglyceride levels while increasing HDL cholesterol [96,97]. It may also protect against diabetic complications such as neuropathy and nephropathy by mitigating nerve damage and reducing kidney injury [98,99].

### 4.4. Anticancer Effects of Rhodiola rosea L. and Its Main Constituents

Cancer is a complex disease when an uncontrolled proliferation and disturbances in cell cycle promote the growth of damaged cells capable to migrate and grow in distant parts of the organism forming tumors there [100,101]. Increased stress, exposure to ultraviolet rays, radiation, pollution, and smoking cause oxidative stress, multiple mutations, chronic inflammation, and apoptosis impairment in the cells [100,102]

Cancer is considered to be a metabolic disease [100,101,103] due to increased aerobic glycolysis [104], increased ROS generation [105], impaired pH regulation leading to acidic environment [106], disturbances in lipid metabolism [107], and enzyme activities [100,108]. These processes lead to increased inflammation [109] and glutamine-driven lipid biosynthesis [110], decreased cardiolipin levels in membranes downregulating enzyme activities [111,112,113], and hyperpolarized mitochondria [100].

**Figure 4:**
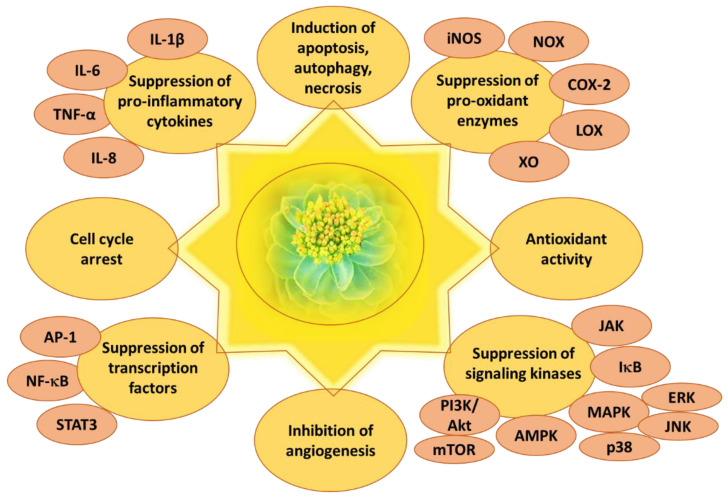
Anticancer effects of *Rhodiola rosea* L. and its bioactive compounds. IL—interleukin; TNF—tumor necrosis factor; AP-1—activator protein 1; NF-κB—nuclear factor kappa-light-chain-enhancer of activated B cells; STAT3—signal transducer and activator 3; iNOS—inducible nitric oxide synthase; NOX—NADPH oxidase; COX-2—cyclooxygenase-2; LOX—lipoxygenase; XO—xanthine oxidase; JAK—Janus kinase; IκB—IκB kinase; MAPK—mitogen activated protein kinase; ERK—extracellular-signal-regulated kinase; JNK—c-Jun N-terminal kinase; p38—p38 kinase; AMPK—AMP-activated protein kinase; PI3K—phosphatidylinositide 3-kinases; Akt—protein kinase B; mTOR—mammalian target of rapamycin.

*R. rosea* and its active constituents have been demonstrated to exert anticancer activity in many cancer models in vitro and in vivo (reviewed recently in [2,4,15,19,31,36,64]). *R. rosea* extracts suppressed tumor growth in vivo [114] by decreasing oxidative stress [31,47,115,116], suppressing inflammation processes [32,117], and activating signaling pathways associated with apoptosis, autophagy, and necrosis [15,18,19,60,118] (Figure 4).

A standardized extract of *R. rosea* Swedish Herbal Rhodiola-5 (SHR-5) and its active compound salidroside (IC_50_ ranged from 70 to 264 μg/mL) inhibited mechanistic target of rapamycin (mTOR), thus suppressing proliferation and upregulating the autophagy processes of human bladder cancer cell lines T24, RT4, 5637, UMUC, and J82 [119]. In HL-60 cells, a 96% ethanol extract of *R. rosea* activated apoptosis and necrosis resulting in cell cycle arrest in G1 phase [59]. In the study of 12 patients, *R. rosea* extracts decreased the recurrence rate of superficial bladder cancer by 50% [120]. Furthermore, *R. rosea* extracts increased the lifespan of yeast and worms, reduced the age-related decline of physical activity and increased stress tolerance [121,122,123], all these effects being important for cancer prevention.

The main active compound of *R. rosea* salidroside is thought to be responsible for its anticancer activity [35,36,39]. The main mechanisms of action of salidroside include suppression of signal transduction pathways PI3k/AKT, JAK/STAT, and MEK/ERK, activation of apoptosis and autophagy, and suppression of inflammation due to inhibition of NF-κB and PI3K/AKT pathways [35,36,39].

Salidroside reduced the release of inflammation-related cytokines, including tumor necrosis factor-α (TNF-α), interleukin (IL)-1β, IL-18, IL-6, cyclooxygenase 2 (COX2), and TGF-β1 and inhibited the nuclear factor kappa-B (NF-κB) signaling pathway in a dose-dependent manner, thus suppressing skin cancer [124]. Furthermore, salidroside inhibited the proliferation and migration of human lung cancer cells through AMPK-dependent NLRP3 inflammasome regulation [125].

Salidroside suppressed the tumorigenesis of HT29 colorectal cells by inhibition of STAT3 and NF-κB signaling pathways, and increased activation of eIF-2α, JNK, and PKR, thus resulting in the upregulation of p53, p38, MAPK, and caspase-8 [126]. Salidroside was more effective than paclitaxel in inhibiting tumor growth in MCF-7 breast cancer cell-bearing nude mice via increasing proapoptotic factor expression and inducing tumor cell apoptosis due to Bcl-2 and p53 downregulation and Bax and caspase 3 upregulation [127]. Salidroside inhibited proliferation, migration, and invasion of human pancreatic cancer PANC1 and SW1990 cells through the inhibition of AKT and ERK signaling pathways [128]. Salidroside induced apoptosis and protective autophagy in human gastric cancer AGS cells through the PI3K/Akt/mTOR pathway [129] and suppressed the growth and invasion of human osteosarcoma cell lines MG63 and U2OS in vitro by inhibiting the JAK2/STAT3 signaling pathway [130]. In HT 1080 fibrosarcoma cells, salidroside regulated the release and expression of MMP-2 and MMP-9 via MAPK signaling pathways [131]. Salidroside inhibited the growth of nasopharyngeal carcinoma xenografts in nude mice by inhibiting of the proliferation and activation of apoptosis acting via targeting miR-4262/GRP78 axis, increasing the level of Bax and decreasing the level of Bcl-2 [132]. Salidroside enhanced doxorubicine sensitivity of HeLa-ADR-luc cells through the regulation of PI3K/Akt/HIF-1α and doxorubicine-induced resistance pathways, and exerted cardioprotective activity [133]. Salidroside suppressed chronic myeloid leukemia cell proliferation and induces apoptosis by regulating the miR-140-5p/wnt5a/β-catenin axis [134], and induced apoptosis in human gastric cancer cells via the downregulation of ENO1/PKM2/GLUT1 expression [135]. Salidroside induces apoptosis and autophagy in human colorectal cancer cells through inhibition of PI3K/Akt/mTOR pathway [136]. Salidroside activated p53, p21Cip1/Waf1, and p16INK4a, thus upregulating the caspase-dependent pathway in ovarian cancer cell lines SKOV3 and A2780 [137]. Furthermore, salidroside inhibited hepatocellular carcinoma metastasis by modulating the activity of the Notch1 signaling pathway [138].

*R. rosea* bioactive compound rosavin exerted protective anti-inflammatory effects in bleomycin-induced pulmonary fibrosis model [139]. It decreased the expression of hydroxyproline and malondialdehyde and enhanced the activities of superoxide dismutase, glutathione peroxidase in lung tissue, upregulated the expression of Nrf2 and downregulated the expression of NF-κB, p65, TGF-β1, and α-SMA [139]. During the investigation of the effects of rosavin on the growth of human Jurkat T cells, apoptosis of splenic mouse CD3 T cells and expression of the surface markers and phosphorylation of extracellular signal-regulated kinase (ERK), it inhibited TNF-related apoptosis-inducing ligand (TRAIL) expression, thus regulating ERK signaling [140]. These results indicate the potential of rosavin to suppress the resistance to apoptosis in autoimmune diseases and cancer [140].

Thus, *R. rosea* and its bioactive constituents exhibit significant anticancer effects, including inhibition of tumor growth, suppression of cell proliferation, induction of apoptosis, antioxidant activity, anti-inflammatory properties, immunomodulation, DNA protection, and chemopreventive potential [18]. However, further research is needed to fully understand the mechanisms and clinical implications of *R. rosea* in cancer treatment and prevention.

## 5. Conclusions and Future Perspectives

Recent research has highlighted the emerging neuroprotective, anti-inflammatory, and anticancer effects of *R. rosea* extract and its main bioactive compounds, encouraging interest in further investigations of its underlying mechanisms of action. Given that mitochondria play a crucial role in cellular energy metabolism and are particularly susceptible to oxidative stress, they can serve as one of the potential biological targets for the main bioactive phenolic compounds found in *R. rosea* root and rhizomes extract.

Notably, *R. rosea* demonstrates a lower toxicity profile while exhibiting stronger stimulating properties compared with ginseng. Moreover, unlike some other adaptogens, *R. rosea*-containing supplements have been found to possess cardioprotective effects, making them suitable for individuals with impaired cardiac function. Consequently, *R. rosea* represents an excellent natural remedy to address situations involving decreased performance, such as fatigue and a sense of weakness, particularly in the context of chronic diseases.

These findings highlight the potential of *R. rosea* and its main bioactive compounds as multifaceted herbal supplements with a range of beneficial effects on the nervous system, inflammation, cancer, and energy metabolism. *R. rosea* investigations have shown promising effects, but there are several shortcomings that need to be addressed. Currently, there is a lack of large-scale, well-controlled clinical trials to establish its safety and efficacy. Standardization of formulations and dosages is lacking, leading to variations in product composition and potency. The exact mechanisms of how *R. rosea* modulates neurotransmitter levels and other physiological processes are not fully understood yet. Long-term effects, potential interactions with medications, and safety for certain populations remain uncertain. Addressing these issues through extensive clinical trials, mechanistic studies, and safety assessments is crucial for better understanding *R. rosea* effects and potential applications.

Future research should focus on large-scale clinical trials with placebo controls to provide robust evidence efficacy and safety of *R. rosea* and its main bioactive compounds. Mechanistic studies at the cellular and molecular levels would help elucidate its physiological actions. Standardization and quality control of *R. rosea* products are essential for consistency and comparability between studies. Safety studies should explore potential interactions and effects on vulnerable populations. Meta-analyses can offer a comprehensive assessment of *R. rosea* effects by pooling data from multiple studies. Thus, further studies are necessary to elucidate the precise mechanisms of action and optimize the use of *R. rosea* and its bioactive compounds in various therapeutic applications.
